# Single Question vs Ongoing Violence Assessment Tool for Intimate Partner Violence Screening

**DOI:** 10.5811/westjem.49021

**Published:** 2026-06-16

**Authors:** Gretchen Nonawzki, Zhengqiu Zhou, Maureen Mohan, Saria Slowey, Donna Carden, Robyn Hoelle

**Affiliations:** * HCA Florida North Florida Hospital, Department of Graduate Medical Education, Gainesville, Florida; † University of Florida, Department of Emergency Medicine, Gainesville, Florida; ‡ University of Central Florida/HCA Florida Healthcare, Gainesville, Florida

## Abstract

**Introduction:**

Intimate partner violence (IPV) represents a pervasive and costly public health issue. Many IPV victims seek care in the emergency department (ED) due to injuries or related medical complaints; yet a substantial number of cases remain undetected. The challenge lies in implementing effective screening tools within the demanding environment of an ED. In this study we aimed to assess the efficacy of two brief IPV screening tools— the single question, “Have you been hit, kicked, punched, or otherwise hurt by someone in the past year?” and the 4-item Ongoing Violence Assessment Tool—and to compare them to the more comprehensive Index of Spouse Abuse, a validated 30-item survey that is considered the gold standard for IPV assessment. Our objective was to identify improved strategies for IPV screening in the busy ED setting.

**Methods:**

In this prospective study, every third English-speaking female patient 18–55 years of age presenting to our ED triage was invited to participate. Each participating patient was sequentially assigned to either the single question or the ongoing assessment screening group. Both groups completed feedback surveys about their experiences and the Index of Spouse Abuse survey. We used chi-square tests to compare categorical variables, and we used frequency tables to calculate the sensitivity, specificity, positive predictive value, and negative predictive value of each IPV screening tool. We compared test characteristics between the Ongoing Violence Asessment Tool or the single question screening tool to the Index of Spouse Abuse using the Fisher exact test. Mean duration was compared using two-tailed Student *t*-tests. The primary outcome was the sensitivity and specificity of the single question assessment. and the 4-item Ongoing Violence Assessment Tool, using the Index of Spouse Abuse as the gold standard for IPV screening. Secondary outcomes were to record the length of time needed to screen with the single question assessment and the Ongoing Violence Assessment Tool and to collect participant feedback on the two screening tools.

**Results:**

A total of 400 participants were enrolled in the study and completed the designated surveys. Screening time was significantly shorter in the single question group (median 6 seconds) compared to the ongoing assessment group (median13 seconds; *P* < .001). Feedback surveys did not reveal significant differences between the two groups. When compared to the Index of Spouse Abuse, the single question tool exhibited sensitivity of 53.3% and specificity of 94.1%, while the ongoing assessment demonstrated slightly higher sensitivity levels of 75.0% and lower specificity of 92.8%. For the single question assesssment, the positive predictive value was 42.1% and negative predictive value was 96.1%. For the Ongoing Violence Assessment Tool, the positive predictive value was 40.1% and the negative predictive value was 98.2%.

**Conclusion:**

The 7-second reduction in overall IPV screening time for the single question compared to the 4-item Ongoing Violence Assessment Tool likely has little clinical benefit, especially with its low sensitivity. Further studies are needed to identify the optimal screening tool for intimate partner violence in the emergency department.

## INTRODUCTION

Intimate partner violence (IPV) is a pervasive healthcare issue that continues to affect millions of U.S. residents annually and disproportionately affects woman.^1^,^2^ The 2016/2017 National Intimate Partner and Sexual Violence Survey demonstrated that 2 in 5 women (51.2 million) have experienced IPV in their lifetime.^3^ Almost six million women in the U.S, reported IPV in the form of contact sexual violence, physical violence, and/or stalking victimization within the 12 months prior to the survey.^3^ To address the challenges in IPV recognition and intervention within the healthcare setting, the Joint Commission for the Accreditation of Healthcare Organizations began requiring policies and procedures for IPV screening in hospitals and clinics in 1992.^4^ However, the efficacy of universal IPV screening remains controversial due to the resources required to execute screening and a lack of evidence that recognizing abuse results in positive outcomes.^5^ Furthermore, instituting successful screening and intervention programs is especially challenging in busy clinical environments like the emergency department (ED) due to time and privacy restraints. To address these challenges, several studies have evaluated the effectiveness of various questionnaires for IPV screening.^6^

Two IPV screening tools previously validated in the ED setting are the single question “Have you been hit, kicked, punched or otherwise hurt by someone in the past year?”^7^ and the four-item Ongoing Violence Assessment Tool.^8^ The single question assessment is a “yes” or “no” answer, with a response of “yes” being considered a positive screen. This question was the most sensitive and specific of the three questions in the previously validated Partner Violence Screen^7^,^9^ and the only one that directly addressed physical violence.^7^ In comparison, the Ongoing Violence Assessment Tool is a 4-item questionnaire with true or false answers. The questionnaire consists of statements that involve physical and emotional aspects of abuse. The items on the survey include the following: 1) “Within the last month my partner has threatened me with a weapon”; 2) “Within the last month my partner has beaten me so badly that I had to seek medical care”; 3) “Within the last month my partner has had no respect for my feelings”; and 4) “Within the last month my partner has acted like he/she would like to kill me.” If a participant answers true to any of these items, then that individual screens positive for IPV. Ernst et al found that the. sensitivity of the Ongoing Violence Assessment Tool in detecting ongoing IPV was 86%, and specificity was 83%.^8^

**Population Health Research Capsule**

What do we already know about this issue?

*Intimate partner violence (IPV) screening in the emergency department (ED) is important for patient safety*.

What was the research question?

*We compared a single question assessment and a 4-item survey to the Index of Spouse Abuse for IPV screening*.

What was the major finding of the study?

*The single question assessment was minimally faster than the 4-item survey (7 seconds) but had lower sensitivity (53% vs 75%)*.

How does this improve population health?

*Additional studies are needed to determine more efficient and effective screening tools for intimate partner violence*.

Developed by Hudson and McIntosh in 1981, the Index of Spouse Abuse (ISA) consists of 30 questions requiring an answer on a 5-point scale from “never” to “very frequently.”^10^ It remains the gold standard for IPV screening and has been evaluated and validated in many different settings to detect both physical and non-physical violence. However, due to the length of time needed to complete an assessment, it does not allow for timely IPV screening in the ED. In this study, we compared the use of a single question tool and a. 4-item ongoing assessment screening tools to the Index of Spouse Abuse, the time required for administration of each of the screening tools, and patient satisfaction with survey administration. We aimed to evaluate the efficacy of brief IPV screening tools for use in the emergency care setting.

## METHODS

### Study Setting and Population

This study was a prospective, single center study in a large, academic ED over a two-month period from May–June 2013. Women 18–55 years of age who presented to the ED for treatment were eligible for inclusion. Exclusion criteria included patients who were medically unstable, non-English speaking, had altered mental status, were unable to be interviewed separately from their partner, had a chief complaint of sexual abuse or sexual assault exam, or were previous enrolled in the study. Every third female presenting to the ED who met the inclusion and exclusion criteria were approached and invited to enroll in the study by a trained research assistant (RA) during the hours that an RA was available.

### Study Design

The RA approached patients in a private treatment room, and visitors/family members were asked to leave for the consent process and survey administrations. If the patient’s visitors declined to leave or re-entered the room and the participant was unable complete the survey in private, that participant was excluded from the study. We did not record the number of patients who were approached, refused, or were unable to separate from their companion; our goal was to protect the patient’s privacy and avoid generating potentially identifiable records in this vulnerable population.

Once alone in a private room, participants were informed about the IPV study and asked to provide verbal consent if they chose to be enrolled. No written consent was collected due to safety concerns. Once patients consented to participate, they were sequentially assigned to either the single question or the 4-question ongoing assessment group. The RA then administered the assigned survey, reading aloud the question (s), and they recorded the participants’ answers using REDCap (Research Electronic Data Capture). This reproduced routine screening processes in triage. We defined “screening time” as the number of seconds needed to administer either assessment. After answering either the single question or the Ongoing Violence Assessment Tool questionnaire, participants of both groups were administered the Evaluation of Screening Tool, which we developed to assesses participants’ comfort with and their opinions regarding the appropriateness of the screening tool they had just experienced. Both groups were then administered the 30-question Index of Spouse Abuse. Both the Evaluation of Screening Tool and the Index of Spouse Abuse were verbally administered by the RA and recorded on REDCap. Any positive answer on any of the screening tools was considered concerning for IPV and triggered the RA to ask the woman’s permission to contact the ED social worker to provide support, which is the standard process when a patient is considered to be experiencing IPV. The RAs were trained to follow a script for recruiting participants, administering the questionnaires, and reacting to positive screens to maintain consistency between participants (Supplemental Document 1). Participants’ baseline demographics were recorded on REDCap during enrollment as well.

### Outcomes

The primary outcome was the sensitivity and specificity of the single question and the 4-item ongoing assessment tools using the Index of Spouse Abuse as the gold standard for IPV screening. Secondary outcomes were time needed to screen with each questionnaire and participants’ feedback on the assessment tools.

### Sample Size

We completed sample size calculations prior to study implementation. To detect a 10% change in positivity rates between the two screening tools, assuming an α of 0.05 and β of 0.2, 200 subjects were needed in each study arm for a total of 400 participants.

### Analysis

We used chi-square to compare categorical variables and frequency tables to calculate the sensitivity, specificity, positive predictive value, and negative predictive value of each IPV screening tool. Test characteristics between the single question and the 4-item ongoing assessment of violence tool were compared to the Index of Spouse Abuse using the Fisher exact test. We compared mean duration using two-tailed Student *t*-tests. Statistical analyses were done with SAS v13.2 (SAS Institute Inc, Cary, NC).

## RESULTS

Over the two-month study period, 400 women participated in this study. For privacy purposes we did not record the number of women who were approached and chose not to participate or stopped participation during the assessment. Of the 400 study participants, 202 were assigned to the single question group and 198 to the 4-itemn assessment for ongoing violence group. Participant demographics are summarized in Table 1. There were no significant differences in age, relationship status, or insurance status between the two groups.The number of participants who screened positive for IPV were similar between the two groups (Table 2). In the single question group, 19 (9.5%) participants had a positive screen for IPV and 182 (90.6%) had a negative screen. In the 4-item ongoing violence assessment group, 24 (12.2%) participants had a positive screen for IPV and 172 (87.8%) had a negative screen . While the screening positivity rates showed no significant difference, there was a significant difference between the time required to complete the surveys. The single question survey took significantly less time, with a median of 6 seconds (interquartile range [IQR] 2) vs the Ongoing Violence Assessment Tool survey with a median of 13 seconds (IQR 16, *P* < .01).After answering the single question or the 4-item ongoing assessment of violence surveys, each participant also completed the Evaluation of Screening Tool consisting of three yes-or-no questions. The first question, “Do you think the screening tool should be given to every patient (men and women) in the emergency room?” was answered “yes” by 180 (89.1%) participants in the single question group and 174 (89.2%) in the Ongoing Violence Assessment Tool group. The second question, “Do you think the screening tool will help patients report violence or abuse that may be occurring at home or with a partner” was answered “yes” by 181 (89.6%) participants in the single question group and 179 (91.8%) in the 4-question Ongoing Violence Assessment Tool group. The final question, “Did you feel uncomfortable answering the screening tool” was answered “yes” by 7 (3.5%) participants in the single question group and 10 (5.1%) in the Ongoing Violence Assessment Tool group. There was no significant difference in feedback survey results between the two groups for any of the Evaluation of Screening Tool questions (Table 3). The Index of Spouse Abuse questionnaire was answered by 394 of 400 study participants. The 30-item questionnaire includes two sections: the ISA-P, which contain questions that screen for physical abuse, and the ISA-NP, which contain questions that screen for nonphysical abuse. Results are considered positive when the ISA-P score is > 10 points or ISA-NP score is greater than 25 points. Thirty-six (9.1%) participants had a positive screen in the ISA-P group, 32 (8.1%) in the ISA-NP group, and 27 (6.9%) participants were positive in both the ISA-P and ISA-NP (Supplemental Table 1). The mean and median scores are also summarized in Supplemental Table 1. Breakdown of responses to individual questions on the Index of Spouse Abuse are summarized in Supplemental Table 2.

When looking at the frequencies of positive and negative screenings using the single question and 4-item ongoing violence assessment compared to the Index of Spouse Abuse, we found they were statistically different. Eight participants were positive for both the single question assessment and the ISA-P and -NP combined; seven were positive for the ISA-P and -NP combined but negative for the single question assessment; 11 were positive for the single question assessment but negative for the ISA-P and -NP combined; and 174 were negative for both the single question assessment and the ISA-P and NP combined (*P* < .001). Nine participants were positive for both the Ongoing Violence Assessment Tool and the ISA-P and -NP combined, three were positive for the ISA-P and -NP combined but negative for the ongoing violence assessment, 13 were positive for the ongoing violence assessment but negative for the ISA-P and -NP combined, and 168 were negative for both the ongoing violence assessment and the ISA-P and -NP combined (*P* < .001, Table 4). Additional details on frequencies of ISA-P and ISA-NP separated and their *P* values are listed in Table 4.

The single question screening tool had sensitivities of 42.9% when compared to the ISA-P, 50.0% when compared to the ISA-NP and 53.3% when compared to the ISA-P and -NP combined. Specificities were 94.4% when compared to the ISA-P, 94.5% when compared to the ISA-NP, and 94.1% when compared to the ISA-P and -NP combined (Table 4). The Ongoing Violence Assessment Tool displayed slightly higher sensitivities—66.7% when compared to the ISA-P, 71.4% when compared to theISA-NP and 75.0% when compared to the ISA-P and NP combined. Specificities of the Ongoing Violence Assessment Tool were similar to the single question survey: 93.3% when compared to both the ISA-P and ISA-NP individually and 92.8% when compared to the ISA-P and -NP combined (Table 4). For the single question survey, positive predictive value ranged between 42.1%–47.4% and negative predictive value was 93.4%–96.1%. For the Ongoing Violence Assessment Tool, positive predictive value was 40.1%–45.5% and negative predictive value was 97.1%–98.2% (Table 4).

Interestingly, when grouped by positive vs negative ISA results, there was no significant difference in the survey evaluation results in the first two questions: 1). “Do you think the screening tool should be given to every patient in the emergency room?”; and 2) “Do you think the screening tool will help patients report violence or abuse that may be occurring at home or with a partner?.” However, the third question “Did you feel uncomfortable answering the screening tool?”, women who screened positive on the ISA more often felt uncomfortable answering the screening tool vs those who did not (Table 5).

## DISCUSSION

Intimate partner violence is an epidemic that affects millions of people every year worldwide.^3^ It is also highly under-recognized, in part due to lack of available, well-validated screening tools that can be completed in a timely manner. In this study we analyzed the efficacy of using a single question survey. and 4-item Ongoing Violence Assessment Tool compared to the ISA, which is the current gold standard for ongoing IPV evaluation. Comparing each violence screening tool to the ISA, we found that both the single question and Ongoing Violence Assessment Tool had adequate specificity, but low sensitivity. The single question tool had higher specificity compared to the Ongoing Violence Assessment Tool, while the ongoing assessment had higher sensitivity. The single questio survey was significantly quicker to administer than the Ongoing Violence Assessment Tool.

Feldhaus et al showed that the three-question Partner Violence Screen achieved a sensitivity of 64.5% and a specificity of 80.3% when compared to the ISA.^7^ In our study, using the single question survey alone, which is one of the three questions from the Partner Violence Screen, we observed lower sensitivity but an increase in specificity compared to the three question Partner Violence Screen. This reduction in sensitivity may be due to the focus only on physical abuse. The single question could potentially miss non-physical IPV, as IPV can also manifest in other forms such as sexual or psychological abuse. Other, more generalized single question screens such as “Do you feel safe at home?” have also been evaluated but demonstrated a sensitivity of only 8.8% and a specificity of 91.2% in primary care settings,^11^ highlighting the ongoing challenge of identifying quick, effective single question IPV screening tools.

When the Ongoing Violence Assessment Tool was previously compared to the Index of Spouse Abuse, it was shown to have a sensitivity of 86% and specificity of 83% in the ED setting.^8^ Our study demonstrates a lower sensitivity at 75% but higher specificity of 93% when combining physical and nonphysical Index of Spouse Abuse items. Numerous factors may have contributed to the differences in the two studies’ results including different patient population, study time frames, and methods of administering the questions. For instance, studies have shown that computer-based screening had higher detection rates of IPV than in person-to-person screenings likely due to a computer‘s increased privacy and added sense of security.^12^ In our study, we chose to simulate actual ED screening circumstances as closely as possible while keeping the participants safety in mind.

This efficacy study demonstrated a significant difference in time to complete the single question vs the Ongoing Violence Assessment Tool. Although the Ongoing Violence Assessment Tool had higher sensitivity, completing it took more than twice as long as the single question (median 13 seconds and 6 seconds, respectively). This may be significant when total ED triage time is expected to be < 5 minutes on average.^13^ However, the clinical significance of a 7-second shorter median screening time should be further evaluated. The American College of Emergency Physicians emphasizes that screening should occur only after initial stabilization and not delay access to care.^14^ The optimal balance between triage efficiency and time spent on screening for various social determinants of health is yet to be determined.^15^

To improve IPV outcomes, increased ED triage screening alone is unlikely to be helpful.^16^, ^17^ Adequate institutional resources such as improved ED workflow, staff training, and the availability of services for patients who screen positive are needed to translate screening into effective care. Programs should include defined referral pathways and institutional capacity to address immediate safety and psychological needs of patients. Improving clinicians’ knowledge of available resources and nonjudgmental communication may improve patient experience and engagement with follow-up, which is crucial for improved outcomes.

## LIMITATIONS

This study has multiple limitations. First, it was conducted at a single site, which may limit generalizability to other clinical settings. Second, only English-speaking women were included. This excludes populations, such as Hispanic communities, which are disproportionately affected by IPV.^18^ Third, the study was restricted to participants 18–55 years of age. Although IPV is less frequently reported among individuals > 55, studies have shown that up to 27.4% of adults > 60 may still experience IPV.^19^ Fourth, sampling only included every third woman due to RA staffing issues and limited space to hold private conversations. Fifth, while the RAs approached approximately 600 women to achieve 400 enrollments, this only represented a 5% sample of the total number presenting to the adult ED at our institution during the study period. This may limit the representativeness of the broader ED population and introduce sampling bias. Sixth, the study did not assess IPV among male patients, despite evidence that > 40% of men in the U.S. report lifetime experiences of IPV.^3^

Further research is needed to determine whether these screening tools perform similarly in other populations, such as men. Seventh, the Evaluation of Screening Tool developed by research personnel has not been validated. Finally, the study was conducted in 2013, which may limit the relevance of the findings to current practice. However, there are still no standard practices for IPV screening and no widely adopted new screening tools.^15^, ^20^ Therefore, updated studies involving broader and more diverse populations are needed to further guide best practices in IPV screening.

## CONCLUSION

Our study showed that while the single question “Have you been hit, kicked, punched, or otherwise hurt by someone in the past year?” minimally decreased ED screening time for intimate partner violence, the question had low sensitivit —missing about half the cases identified by the Index of Spouse Abuse. The 4-item Ongoing Violence Assessment Tool had slightly higher sensitivity than the single question. However, given the low sensitivities of both screening tools, their utility as screening strategies for detecting IPV in the ED setting must be further evaluated. The 7-second shorter screening time for the single question survey, as compared to the Ongoing Violence Assessment Tool, likely has little clinical relevance, especially given the single question’s sub-optimal sensitivity for detecting IPV. Physicians should be made aware of the potential for the under-recognition of IPV cases with these rapid screening tools and remain vigilant for any signs of partner abuse.

## Supplementary Information







## Figures and Tables

**Figure:**
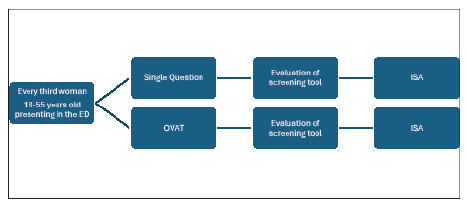
Sequence of questionnaires conducted in a study of intimate partner violence screening tools. *OVAT*, Ongoing Violence Assessment Tool; *ISA*, Index of Spouse Abuse.

**Table 1:** Characteristics of patients undergoing screening for intimate partner violence using either the single question tool or the 4-question Ongoing Violence Assessment Tool.

Characteristics	Total study population N = 400 (%)	Single Question n = 202 (%)	Ongoing Violence Assesssment Tool n = 198 (%)	*P* value
Age, years				
Mean (SD)	34.8 (11.4)	35.3 (11.7)	34.3 (11.1)	.35
Median (IQR)	34.0 (22.0)	34.0 (23.0)	34.0 (19.0)	
Relationship status				
Single	108 (27.5)	49 (24.5)	59 (30.6)	.13
Relationship (living with partner)	85 (21.6)	49 (24.5)	36 (18.7)	
Relationship (not living with partner)	86 (21.9)	38 (19.0)	48 (24.9)	
Married	114 (29.0)	64 (32.0)	50 (25.9)	
Insurance				
None	106 (27.0)	54 (27.0)	52 (26.9)	.76
Public (Medicare/Medicaid)	155 (39.4)	82 (41.0)	73 (37.8)	
Private	132 (33.6)	64 (32.0)	68 (35.2)	

*SD*, standard deviation; *IQR*, interquartile range.

**Table 2:** Intimate partner violence screening outcomes using the single question and 4-item Ongoing Violence Assessment Tools.

		Positive (%)	Negative (%)
Single Question, n = 201			
1.	Have you been hit, kicked, punched or otherwise hurt by someone in the past year?	19 (9.5)	182 (90.6)
	Positive = “True”		
	Negative = “False”		
Ongoing Violence Assessment Tool, n = 196			
1.	Within the last month my partner has threatened me with a weapon.	0.0 (0.0)	196 (100.0)
	Positive = “True”		
	Negative = “False”		
2.	Within the last month my partner has beaten me so badly that I had to seek medical care.	2 (1.0)	194 (99.0)
	Positive = “True”		
	Negative = “False”		
3.	Within the last month my partner has had no respect for my feelings.	23 (11.7)	173 (88.3)
	Positive = “Occasionally” or more		
	Negative = “Rarely” or “Never”		
4.	Within the last month my partner has acted like he/she would like to kill me.	3 (1.5)	193 (98.5)
	Positive = “True”		
	Negative = “False”		
Overall Screening Results for the Ongoing Violence Assessment Tool:		24 (12.2)	172 (87.8)

**Table 3:** Outcomes of participant experience survey (Evaluation of Screening Tool) in participants assessed by the single question or 4-item Ongoing Violence Assessment Tool for intimate partner violence screening.

	Single Question (n = 202)		Ongoing Violence Assessment Tool (n = 198)		

	Yes (%)	No (%)	Yes (%)	No (%)	*P* value
1. Do you think the screening tool should be given to every patient (men and women) in the emergency room?	180 (89.1)	22 (10.9)	174 (89.2)	21 (10.8)	.97
2. Do you think the screening tool will help patients report violence or abuse that may be occurring at home or with a partner?	181 (89.6)	21 (10.4)	179 (91.8)	16 (8.2)	.45
3. Did you feel uncomfortable answering the screening tool?	7 (3.5)	193 (96.5)	10 (5.1)	185 (94.9)	.46

**Table 4:** Test characteristics of the single question survey and the 4-item Ongoing Violence Assessment Tool in comparison to the 30-item Index of Spouse Abuse.

		Single Question vs Index of Spouse Abuse (n = 201)						

		Positive	Negative	Sensitivity	Specificity	PPV	NPV	*P* value
ISA-P	Positive	9	12	42.9%	94.4%	47.4%	93.4%	< .001
	Negative	10	169					
ISA-NP	Positive	9	9	50.0%	94.5%	47.4%	95.0%	< .001
	Negative	10	172					
ISA-P + ISP-NP	Positive	8	7	53.3%	94.1%	42.1%	96.1%	< .001
	Negative	11	174					

		Ongoing Violence Assessment Tool vs Index of Spouse Abuse (n = 196)						

		Positive	Negative	Sensitivity	Specificity	PPV	NPV	*P* value
		
ISA-P	Positive	10	5	66.7%	93.3%	45.5%	97.1%	< .001
	Negative	12	166					
ISA-NP	Positive	10	4	71.4%	93.3%	45.5%	97.7%	< .001
	Negative	12	167					
ISA-P + ISP-NP	Positive	9	3	75.0%	92.8%	40.1%	98.2%	< .001
	Negative	13	168					

*ISA*, Index of Spouse Abuse; *ISA-P*, Index of Spouse Abuse physical; *ISA-NP*, Index of Spouse Abuse nonphysical; *PPV*, positive predictive value; *NPV*, negative predictive value.

**Table 5:** Participants’ assessment of the single question survey and the 4-item Ongoing Violence Assessment Tool using the Evaluation of Screening Tool results separated by Index of Spouse Abuse results.

		Yes (%)	No (%)	*P* value
1. Do you think the screening tool should be given to every patient in the emergency room?				
ISA-P	Positive	32 (88.9)	4 (11.1)	.78
	Negative	321 (89.7)	37 (10.3)	
ISA-NP	Positive	28 (87.5)	4 (12.5)	.76
	Negative	325 (89.8)	37 (10.2)	
ISA-P + ISP-NP	Positive	24 (88.9)	3 (11.1)	.75
	Negative	329 (89.7)	38 (10.4)	
2. Do you think the screening tool will help patients report violence or abuse that may be occurring at home or with a partner?				
ISA-P	Positive	31 (86.1)	5 (13.9)	.35
	Negative	328 (91.6)	30 (8.4)	
ISA-NP	Positive	28 (87.5)	4 (12.5)	.51
	Negative	331 (91.4)	31 (8.6)	
ISA-P + ISP-NP	Positive	23 (85.2)	4 (14.8)	.28
	Negative	336 (91.6)	31 (8.5)	
3. Did you feel uncomfortable answering the screening tool?				
ISA-P	Positive	5 (13.9)	31 (86.1)	< .001
	Negative	10 (2.8)	346 (97.2)	
ISA-NP	Positive	4 (12.5)	28 (87.5)	.03
	Negative	11 (3.1)	349 (96.9)	
ISA-P + ISP-NP	Positive	4 (14.8)	23 (85.2)	.02
	Negative	11 (3.0)	354 (97.0)	

*ISA-P*, Index of Spouse Abuse physical; *ISA-NP*, Index of Spouse Abuse nonphysical.
