# Supplementary material for: Single Question vs Ongoing Violence Assessment Tool for Intimate Partner Violence Screening
Source: West J Emerg Med. 2026 Jun 16;27(4):977–84. doi: 10.5811/westjem.49021 (PMC13436654; doi:10.5811/westjem.49021)
Supplement: Supplementary file 1 [file wjem-27-977-s001.docx]

| Supplemental table 1. Summary of index of spouse abuse (ISA) results of all participants that underwent single question (SQ) and ongoing violence assessment tool (OVAT) for intimate partner violence screening | |
| --- | --- |
| Characteristics | Total Study  Population (n=394, %) |
| Index of Spouse Abuse-Physical (ISA-P) |  |
| Positive (ISA-P ≥ 10) | 36 (9.1) |
| Negative (ISA-P < 10) | 358 (90.9) |
| Mean Score (SD) | 3.5 (11.8) |
| Median Score (IQR) | 0.0 (0.0) |
| Index of Spouse Abuse-Non Physical (ISA-NP) |  |
| Positive (ISA-P ≥ 25) | 32 (8.1) |
| Negative (ISA-P < 25) | 362 (91.9) |
| Mean Score (SD) | 5.7 (15.6) |
| Median Score (IQR) | 0.0 (2.0) |
| Combined ISA-P and ISA-NP Scales |  |
| Both Positive (ISA-P and ISA-NP) | 27 (6.9) |
| One Positive or Neither Positive | 367 (93.2) |

SD – standard deviation, IQR – interquartile range
