# Supplementary material for: Single Question vs Ongoing Violence Assessment Tool for Intimate Partner Violence Screening
Source: West J Emerg Med. 2026 Jun 16;27(4):977–84. doi: 10.5811/westjem.49021 (PMC13436654; doi:10.5811/westjem.49021)
Supplement: Supplementary file 2 [file wjem-27-977-s002.docx]

Supplemental table 2. Participants responses to Index of Spouse Abuse (ISA) separated by individual questions (n=394).

|  | Never (%) | Rarely (%) | Occasionally (%) | Frequently (%) | Very Frequently (%) |
| --- | --- | --- | --- | --- | --- |
| 1. My partner belittles me. | 342 (86.36) | 23 (5.81) | 10 (2.53) | 13 (3.28) | 8 (2.02) |
| 2. My partner demands obedience to his whims. | 354 (89.39) | 14 (3.54) | 9 (2.27) | 10 (2.53) | 9 (2.27) |
| 3. My partner becomes surly and angry if I tell him he is drinking too much. | 353 (89.14) | 8 (2.02) | 16 (4.04) | 8 (2.02) | 11 (2.78) |
| 4. My partner makes me perform sex acts that I do not enjoy or like. | 385 (97.22) | 2 (0.51) | 2 (0.51) | 3 (0.76) | 4 (1.01) |
| 5. My partner becomes very upset if dinner, housework or laundry is not done when he thinks it should be. | 356 (89.90) | 16 (4.04) | 11 (2.78) | 6 (1.52) | 7 (1.77) |
| 6. My partner is jealous and suspicious of my friends. | 322 (81.31) | 21 (5.30) | 22 (5.56) | 8 (2.02) | 23 (5.81) |
| 7. My partner punches me with his fists. | 380 (95.96) | 6 (1.52) | 3 (0.76) | 4 (1.01) | 3 (0.76) |
| 8. My partner tells me I am ugly and unattractive. | 380 (95.96) | 4 (1.01) | 5 (1.26) | 2 (0.51) | 5 (1.26) |
| 9. My partner tells me I really couldn't manage or take care of myself without him. | 350 (88.38) | 8 (2.02) | 15 (3.79) | 6 (1.52) | 17 (4.29) |
| 10. My partner acts like I am his personal servant. | 360 (90.91) | 8 (2.02) | 7 (1.77) | 13 (3.28) | 8 (2.02) |
| 11. My partner insults or shames me in front of others. | 357 (90.15) | 10 (2.53) | 15 (3.79) | 1 (0.25) | 13 (3.28) |
| 12. My partner becomes very angry if I disagree with his point of view. | 332 (83.84) | 21 (5.30) | 22 (5.56) | 10 (2.53) | 11 (2.78) |
| 13. My partner threatens me with a weapon. | 385 (97.47) | 2 (0.51) | 6 (1.52) | 2 (0.51) | 0 (0.0) |
| 14. My partner is stingy in giving me enough money to run our home. | 364 (92.15) | 9 (2.28) | 6 (1.52) | 2 (0.51) | 14 (3.54) |
| 15. My partner belittles me intellectually. | 368 (93.16) | 6 (1.52) | 8 (2.03) | 4 (1.01) | 9 (2.28) |
| 16. My partner demands that I stay home to take care of the children. | 380 (96.20) | 3 (0.76) | 4 (1.01) | 4 (1.01) | 4 (1.01) |
| 17. My partner beats me so badly that I must seek medical help. | 387 (97.97) | 2 (0.51) | 3 (0.76) | 1 (0.25) | 2 (0.51) |
| 18. My partner feels that I should not work or go to school. | 371 (93.92) | 7 (1.77) | 7 (1.77) | 4 (1.01) | 6 (1.52) |
| 19. My partner is not a kind person. | 354 (89.85) | 14 (3.55) | 15 (3.81) | 4 (1.02) | 7 (1.78) |
| 20. My partner does not want me to socialize with my female friends. | 352 (89.34) | 11 (2.79) | 12 (3.05) | 7 (1.78) | 12 (3.05) |
| 21. My partner demands sex whether I want it or not. | 374 (94.92) | 3 (0.76) | 5 (1.27) | 6 (1.52) | 6 (1.52) |
| 22. My partner screams and yells at me. | 328 (83.25) | 21 (5.33) | 16 (4.06) | 10 (2.54) | 19 (4.82) |
| 23. My partner slaps me around my face and head. | 376 (95.43) | 6 (1.52) | 3 (0.76) | 4 (1.02) | 5 (1.27) |
| 24. My partner becomes abusive when he drinks. | 365 (92.64) | 9 (2.28) | 7 (1.78) | 5 (1.27) | 8 (2.03) |
| 25. My partner orders me around. | 350 (88.83) | 14 (3.55) | 10 (2.54) | 10 (2.54) | 10 (2.54) |
| 26. My partner has no respect for my feelings. | 337 (85.53) | 12 (3.05) | 22 (5.58) | 4 (1.02) | 19 (4.82) |
| 27. My partner acts like a bully towards me. | 355 (90.10) | 8 (2.03) | 9 (2.28) | 10 (2.54) | 12 (3.05) |
| 28. My partner frightens me. | 360 (91.37) | 7 (1.78) | 7 (1.78) | 7 (1.78) | 13 (3.30) |
| 29. My partner treats me like a dunce. | 357 (90.61) | 4 (1.02) | 14 (3.55) | 7 (1.78) | 12 (3.05) |
| 30. My partner acts like he would like to kill me. | 373 (94.67) | 4 (1.02) | 6 (1.52) | 4 (1.02) | 7 (1.78) |

Score on ISA: Never – 1, Rarely – 2, Occasionally – 3, Frequently – 4, Very frequently – 5
