# Supplementary material for: Single Question vs Ongoing Violence Assessment Tool for Intimate Partner Violence Screening
Source: West J Emerg Med. 2026 Jun 16;27(4):977–84. doi: 10.5811/westjem.49021 (PMC13436654; doi:10.5811/westjem.49021)
Supplement: Supplementary file 3 [file wjem-27-977-s003.docx]

Supplemental document 1: Research assistant script for participant screening and survey administration

1. Identify every third female (18-55yo) from list that the pivot nurse is creating.
   a. Patients are excluded if: medically unstable, non-English speaking, have impaired mental status, will not separate from their partner, present for sexual abuse or sexual assault exam, or have already enrolled in the study.
2. Ask for participation
   a. Approach patient after triage is completed
   b. *“Ms. _________? Hello, my name is _______. I am a research assistant and was wondering if you would be interested in taking a survey about women’s health? This survey will take about 30 minutes.”*
3. Separate participants from visitors
   a. *“Ms. ________, thank you for agreeing to take the survey. In order to have all the participants take the survey in the same way I can only give you the survey in complete privacy and your visitors can not be in the room. Do you mind if they step out?”*
   b. If there is ANY reason the participant can not be separated from her visitors then she is excluded from the study and you can thank her for her consideration and excuse yourself.
4. Informed Consent
   a. *“Now that you are alone, I need to ask you if you would be willing to participate in a survey about Intimate Partner Violence also known as domestic violence instead of women’s health. I am sorry I could not ask you this before, but for our participant’s safety we must wait until they are alone. Do you still want to participate?”*
   b. *“Ms. _______, I am going to show you this letter about the study we are asking you to participate in and if you have any questions I would be happy to answer them.”*
   c. Read the letter to them and answer any questions they may have.
5. Survey Administration
   a. Every other participant will be assigned to either the SQ or the OVAT. See diagram. RedCap will do this for you.
   b. Survey and the rest of the information you need will be on REDCap online database.
   c. You will read each question to them and record their answer in the REDCap database. Start the stop watch at the start of the screening tool portion and stop when you are done with that portion. This is the time we want recorded.
   d. You will administer the following questionnaires as guided by the REDCap database:

Will she participate yes or no (if not, why not)?

Screening Tool [SQ(1) versus OVAT(4)]

Evaluation of the Screening Tools Portion (5)

ISA (30)

Demographics (4)

e. With any positive answer to any questions of the three IPV screening tools, the RA will be instructed to offer assistance to the participant using the following script exactly.

*“You have answered some of the questions I asked you in a way that indicates you may be experiencing intimate partner violence. Can I call our social worker and have her speak to you?”*

If the woman would like to speak to someone then you need to find the patient’s nurse and notify them. You are to strictly stick to this script. You are not in a position to offer advice, condolences, empathy, or any other intervention. This is very important for your safety and the participant’s safety. If the participant does not want you to contact anyone, then thank her for her time and excuse yourself.

6. If the patient at any time wants to withdraw from the study, she is allowed and you will erase her information from the database at that time or contact Dr. Hoelle or the research coordinator and we can do it as well.
7. After filling in ALL items required in REDCap. Please ask the participant if she has any questions and offer to go and inform her visitors that she is done.
8. Make sure you have saved the data appropriately.
9. At the end of your shift please return the computer to the attending office.
